# Prediction of Treatment Response in Hepatitis B and Hepatitis C Coinfected Patients Using a Leakage‐Proof, Internally Validated Logistic Regression Pipeline

**DOI:** 10.1155/av/2578085

**Published:** 2026-07-09

**Authors:** Eisha Hamid, Ayesha Malik, Momna Arooj Malik, Maleha Asim, Javed Ashraf

**Affiliations:** ^1^ Riphah International University, Islamabad, Pakistan, riphah.edu.pk; ^2^ Riphah International University Gulberg Green Campus, Islamabad, Pakistan; ^3^ Dental Public Health, Health Services Academy, Islamabad, Pakistan, hsa.edu.pk

**Keywords:** antiviral agents, coinfection, hepatitis B, hepatitis C, treatment outcome

## Abstract

Coinfection of hepatitis B virus and hepatitis C virus presents great difficulty in treatment procedures, especially in terms of prediction of the response to the direct‐acting antiviral therapy. In view of this, developing adequate prediction models would play a vital role in ensuring better personalization and effectiveness in terms of treatment. The objective of this paper is to develop and validate prediction models for the identification of patients’ responses to direct‐acting antiviral therapy in chronic HBV/HCV coinfected patients following treatment completion. This retrospective clinical prediction study involved the use of medical records of 154 patients with HBV/HCV coinfection treated with the help of direct‐acting antiviral therapy in Holy Family Hospital, Rawalpindi, Pakistan. Sixteen predictors were used to develop logistic regression models to predict treatment responses at 4 and 12 weeks. In addition, to counter the problems of class imbalance, the synthetic minority oversampling technique was applied to the datasets, while nested stratified cross‐validation was used for hyperparameter tuning and model validation. Performance was evaluated through different performance metrics such as ROC–AUC, accuracy, precision, recall, and F1‐score. The performance of the two models was very good at each endpoint. For the endpoint of 4 weeks, the ROC–AUC score was 0.858 (95% CI 0.746–0.942), the accuracy was 0.760, and the F1‐score was 0.851. In the case of the 12‐week endpoint, the ROC–AUC score was 0.850 (95% CI 0.769–0.916), the accuracy was 0.786, and the F1‐score was 0.856. The important predictors were HCV genotypes, age, body mass index, hemoglobin, and liver function test results. Good model calibration was evident from the calibration graphs, which showed slight deviation from the ideal calibration line at both endpoints. The current study developed prediction models for treatment response in HBV/HCV coinfected patients based on clinical and laboratory information from baseline. The models showed very good internal validity, but the 12‐week model performed slightly better than the 4‐week model in terms of classification balance. Such models represent useful tools for decision‐making in case of a personalized approach.

## 1. Introduction

**FIGURE 1:**
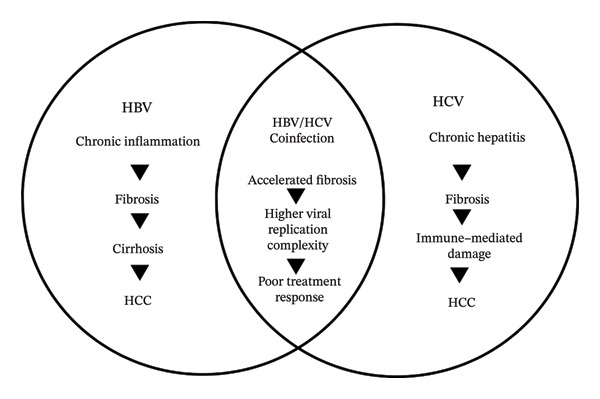
Pathological interaction and overlapping disease mechanism of HBV/HCV coinfection.

Viral hepatitis remains a major health problem worldwide, which results in morbidity and mortality, especially through chronic infection, liver cirrhosis, and hepatocellular carcinoma associated with hepatitis B virus (HBV) and hepatitis C virus (HCV) infection. In 2022, there was an increase in mortality from 1.1 million cases recorded in 2019 to 1.3 million. Among the 1.3 million deaths, 83% resulted from HBV infection, while 17% resulted from HCV infection [1]. Research has revealed that liver cirrhosis patients who suffer from HBV infection have a high risk of inpatient death than those suffering from HCV [2]. The dual infection of HCV and HBV is an important worldwide phenomenon, and its incidence varies according to the geographical location. Since both viruses have similar transmission routes (bloodborne), coinfection is especially frequent in endemic areas [3]. Globally, 1%–15% of patients with HBV infection are coinfected with HCV [4]. In Pakistan, 12.5% of HBV‐infected patients are also coinfected with HCV. Coinfection of HBV with HCV accelerates the progression of liver fibrosis and increases the incidence of liver cirrhosis and hepatocellular carcinoma compared to HCV mono‐infection [5]. Figure 1 provides a schematic presentation of the complex pathology and shared behavior of HBV/HCV coinfection.

Usually, traditional statistical methods have been used to analyze the demographic and clinical risk factors for coinfections concerning HBV and HCV [6]. Nevertheless, these methods are limited in scope, do not cater to selection bias, and are unable to handle complex and nonlinear associations and patterns in data.

Artificial intelligence (AI), especially machine learning (ML), offers a robust solution for these issues. Recent studies have highlighted the importance of AI mechanisms to better understand viral mechanisms and offer therapeutic targeting [7]. While ML models are capable of recognizing complicated patterns within clinical data with no assumptions made prior [8], the logistic regression (LR) model is still considered the preferred model in clinical research for the high performance of the algorithm along with established validation methodology. The increasing use of the LR model in clinical medicine involves diagnostic modeling, prediction of risk factors, and prognostic evaluation, making it a cornerstone of clinical modeling practice [9, 10].

The objective of the current study was to develop and internally validate a leakage‐proof LR for the prediction of response to treatment by HCV and HBV using the PCR test at Weeks 4 and 12.

## 2. Materials and Methods

### 2.1. Study Design and Setting

This retrospective clinical prediction study used routinely collected clinical and laboratory data from patients with HBV and HCV coinfection who completed direct‐acting antiviral therapy at liver clinics of Holy Family Hospital, Rawalpindi, Pakistan. The objective was to develop and internally validate endpoint‐specific prediction models for treatment responses at 4 weeks and 12 weeks using baseline pretreatment variables only.

### 2.2. Study Population and Eligibility

The final analytic cohort comprised 154 adult patients with HBV/HCV coinfection. Patients were eligible if they were aged 18 years or older and had chronic HBV/HCV coinfection according to the study recruitment criteria. Patients with liver decompensation, HIV or other hepatitis‐virus coinfections, hepatocellular carcinoma, pregnancy, severe renal impairment, extrahepatic malignancy not in remission, or documented autoimmune, metabolic, or genetic liver disease were excluded.

### 2.3. Ethical Approval

Written informed consent was obtained from all participants before enrollment. The study was approved by the Ethics Committee/Institutional Review Committee of Riphah International University (Approval No. IIDC/IRC/2024/011/002) and complied with the Declaration of Helsinki.

### 2.4. Data Source and Variables

All final manuscript‐grade analyses were performed exclusively on the original patient dataset. In total, there were 154 cases for modeling, with 18 variables being considered, and no missing or duplicated data were present at the stage of performing the analysis. As endpoints, two response variables were modeled: treatment response at 4 weeks and treatment response at 12 weeks. According to the dataset, 4‐week and 12‐week endpoints were identified as responders and nonresponders. In turn, for modeling purposes, both endpoints were coded into binary form: responder = 1, nonresponder = 0. Overall, 16 numerical variables were used as predictors in this project: albumin, hemoglobin, ALP, ALT, AST, HCV, HBV, APRI score, FIB‐4 score, BMI, age, and bilirubin. In addition, categorical variables—gender, interferon treatment history, HCV genotype, and IL28 B category—were considered predictors. Target leakage avoidance required excluding both posttreatment outcome variables each time one of the endpoints was modeled.

### 2.5. Descriptive Analysis

For each endpoint, responder and nonresponder groups’ baseline characteristics were presented separately. Continuous data were represented by means and standard deviations, whereas categorical data were described by frequencies and percentages. The independent *t*‐test or Mann–Whitney test and chi‐square or Fisher’s exact test were performed to compare the two groups. All of the above statistical methods were used for descriptive purposes only and were not included in the feature selection process based on the outcome.

### 2.6. Preprocessing and Leakage Control

All preprocessing was done using a leakage‐free preprocessing pipeline. Numerical columns were preprocessed through median imputation and then standardized. Categorical columns were preprocessed with most frequent imputation and one‐hot encoding with *handle unknown* set to ignore. Even though there were no missing values in the original dataset, preprocessing through imputation was still included in the preprocessing pipeline so that a consistent preprocessing methodology could be maintained. Parameters for all preprocessors were only learned on the training set.

### 2.7. Handling of Class Imbalance

Unbalanced response classes especially after 4 weeks were solved by applying the synthetic minority oversampling technique (SMOTE). SMOTE was implemented in the *imblearn* pipeline and thus was used only for training sets and not for validation sets. The actual distributions of classes after these endpoints included 142 responders and 12 nonresponders after 4 weeks and 127 responders and 27 nonresponders after 12 weeks.

### 2.8. Model Specification

The final confirmatory classifier was regularized LR. This decision reflected the corrected leakage‐proof workflow and the need for a transparent, stable classifier under a limited sample size. The model used the *liblinear* solver with *max_iter* = 5000 and a fixed random seed of 42. Hyperparameter tuning was performed over the following grid: LR regularization strength C in {0.01, 0.1, 1, 10} and SMOTE neighborhood size *k_neighbors* in {2, 3, 5}.

### 2.9. Internal Validation

Nested stratified cross‐validation was used to separate model tuning from unbiased performance estimation. For the 4‐week endpoint, the model was evaluated using outer 5‐fold stratified cross‐validation and inner 3‐fold stratified cross‐validation for hyperparameter tuning. For the 12‐week endpoint, the model was evaluated using outer 3‐fold stratified cross‐validation and inner 2‐fold stratified cross‐validation for hyperparameter tuning. At both endpoints, the outer‐fold data remained untouched until final evaluation. All reported performance metrics were derived from pooled out‐of‐fold predictions.

### 2.10. Performance Metrics

Performance was evaluated using area under the receiver operating characteristic curve (ROC–AUC), accuracy, precision, recall, F1‐score, and Brier score. Aggregated out‐of‐fold confusion matrices were also computed for each endpoint. Ninety‐five percent confidence intervals for ROC–AUC were estimated by bootstrap resampling of pooled out‐of‐fold predictions using 2000 bootstrap replicates.

### 2.11. Calibration Analysis

The assessment of calibration was done through reliability plots that were derived from the predicted probability obtained out‐of‐fold. These plots were used to examine the agreement between predicted probabilities and observed event frequencies at both endpoints.

### 2.12. Stability Analysis

Because the minority class was sparse, especially at 4 weeks, a learning‐curve style stability analysis was performed using repeated stratified hold‐out resampling at training fractions of 0.60 and 0.85. Mean AUC, standard deviation, and 95% confidence intervals around the mean were summarized across repeated splits. For this auxiliary analysis, the SMOTE neighborhood grid was restricted to feasible values for sparse‐event settings to avoid invalid oversampling within very small inner folds.

### 2.13. Model Interpretability

To facilitate interpretation, a separate set of LR models was fitted for each endpoint of 4 week and 12 week, respectively, using the hyperparameters selected during cross‐validation. The standardized coefficients from the models were then considered for directional interpretation of predictor effects.

### 2.14. Software and Reproducibility

All analysis was performed in Python with the use of pandas, NumPy, matplotlib, scikit‐learn, and imbalanced‐learn libraries, all running in a Jupyter Notebook (Jupyter code 5) (Supporting File S1) with a fixed random seed of 42. All reproducibility materials contained the code used to perform the analysis in an executable notebook format, summary table exports, and environment setup files. The earlier balanced datasets were not used for final model development and were retained only as audit artifacts.

## 3. Results

Table 1 presents the characteristics of the treatment responders and nonresponders at the 4‐week endpoint, focusing on demographic parameters (HCV genotype, sex, and age) and biochemical markers (AST, FIB‐4 score, bilirubin, APRI score, HBV‐DNA, BMI, HCV‐RNA, ALT, IL‐28 B genotype, hemoglobin, albumin, and ALP). In regard to the characteristics of responders (*n* = 142) and nonresponders (*n* = 12), the mean age of the former group was 50.08 ± 11.70 years, and the mean BMI was 25.39 ± 2.50 kg/m^2^. Notably, the mean age of the latter group was higher than that of the former. Statistical significance in relation to the HCV genotype was established (*p* < 0.001). It has been shown that Genotype 3 was prevalent in 85.9% of cases of responders. Nonresponders showed a significant elevation in AST, ALT, and bilirubin levels (*p* < 0.05), which suggested ongoing hepatic injury. However, there were no significant differences in liver function parameters, including ALP (*p* = 0.303) and albumin (*p* = 0.523), between the two groups, indicating limited prediction potential for an early therapeutic response.

**TABLE 1:** Biochemical and clinical parameters and genotype distribution in treatment response at 4 Weeks.

Variables	Responders (*n* = 142)	Nonresponders (*n* = 12)	*p* value
ALT (U/L)	53.72 ± 18.62	69.92 ± 21.08	< 0.05
AST (U/L)	45.23 ± 23.01	68.67 ± 23.15	< 0.05
ALP (U/L)	80.06 ± 19.73	89.33 ± 26.76	0.303
Albumin (g/dL)	4.31 ± 0.53	4.17 ± 0.58	0.523
Bilirubin (mg/dL)	0.80 ± 0.27	1.03 ± 0.11	< 0.05
FIB‐4 score	1.77 ± 0.67	2.43 ± 0.74	< 0.001
APRI score	0.66 ± 0.35	0.97 ± 0.22	< 0.001

Genotype distribution			
Genotype 3	85.9%	25.0%	< 0.001
Non‐genotype 3	14.1%	75.0%	< 0.001

*Note: n*, number of patients; ALT, alanine aminotransferase; AST, aspartate aminotransferase; ALP, alkaline phosphatase; FIB‐4, fibrosis‐4; statistical significance was set as *p* < 0.05.

Abbreviation: APRI, AST to Platelet Ratio Index.

Table 2 compares the clinical and laboratory variables between responders (*n* = 127) and nonresponders (*n* = 27) at the 12‐week treatment endpoint. The analysis revealed more pronounced differences in the 12‐week endpoint than the 4‐week endpoint. The liver enzymes ALT and AST remained significantly elevated among the nonresponders (*p* < 0.05), suggesting persistent hepatic dysfunction. A significant association with treatment response was also observed for the HCV genotype (*p* < 0.001), with 89% of the responders having Genotype 3. Unlike the 4‐week results, both albumin (*p* = 0.003) and ALP (*p* = 0.004) were statistically significant at Week 12. High albumin was related to a positive therapeutic response, while high ALP was linked to a negative therapeutic response.

**TABLE 2:** Biochemical and clinical parameters and genotype distribution in treatment response at 12 Weeks.

Variables	Responders (*n* = 127)	Nonresponders (*n* = 27)	*p* value
ALT (U/L)	53.72 ± 18.62	69.92 ± 21.08	< 0.05
AST (U/L)	45.23 ± 23.01	68.67 ± 23.15	< 0.05
ALP (U/L)	80.06 ± 19.73	89.33 ± 26.76	0.0044
Albumin (g/dL)	4.36 ± 0.51	4.00 ± 0.55	0.0036
Bilirubin (mg/dL)	0.80 ± 0.27	1.03 ± 0.11	0.0029
FIB‐4 score	1.77 ± 0.67	2.43 ± 0.74	< 0.001
APRI score	0.66 ± 0.35	0.97 ± 0.22	< 0.001

Genotype distribution			
Genotype 3	89.0%	55.6%	< 0.001
Non‐genotype 3	11.0%	44.4%	< 0.001

*Note: n*, number of patients; ALT, alanine aminotransferase; AST, aspartate aminotransferase; ALP, alkaline phosphatase; FIB‐4, fibrosis‐4; Statistical significance was set at *p* < 0.05.

Abbreviation: APRI, AST to Platelet Ratio Index.

Fibrosis markers, including APRI and FIB‐4 scores, remained significantly elevated in nonresponders at both 4‐week and 12‐week treatment intervals (*p* < 0.001), indicating their potential role as reliable indicators of poor treatment response.

At 4 weeks, the internally validated LR pipeline achieved a pooled out‐of‐fold ROC–AUC of 0.858 with a bootstrap 95% confidence interval of 0.746–0.942. Classification performance included an accuracy of 0.760, precision of 0.991, recall of 0.746, F1‐score of 0.851, and Brier score of 0.176. At 12 weeks, the corresponding values were ROC–AUC 0.850 (95% confidence interval 0.769–0.916), accuracy 0.786, precision 0.961, recall 0.772, F1‐score 0.856, and Brier score 0.174. Both endpoint‐specific models demonstrated good internal discrimination. The 12‐week model showed modestly better classification balance, whereas the 4‐week model achieved slightly higher discrimination but under more extreme class imbalance as shown in Table 3.

**TABLE 3:** Out‐of‐fold predictive performance for Week 4 and Week 12 endpoints.

Endpoint	Total (*N*)	Positive class	Negative class	OOF AUC	95% CI for AUC	Accuracy	Precision	Recall	F1‐score	Brier score
Week 4	154	142	12	0.858	0.746 to 0.942	0.760	0.991	0.746	0.851	0.176
Week 12	154	127	27	0.850	0.769 to 0.916	0.786	0.961	0.772	0.856	0.174

The aggregated out‐of‐fold confusion matrices for both endpoints are presented in Table 4. The confusion matrices indicate very high precision for positives at both endpoints; however, they also indicate some misses on the part of responder instances, suggesting a cautious approach due to imbalance.

**TABLE 4:** Aggregated out‐of‐fold confusion matrices.

Endpoint	TN	FP	FN	TP
Week 4	11	1	36	106
Week 12	23	4	29	98

**FIGURE 2:**
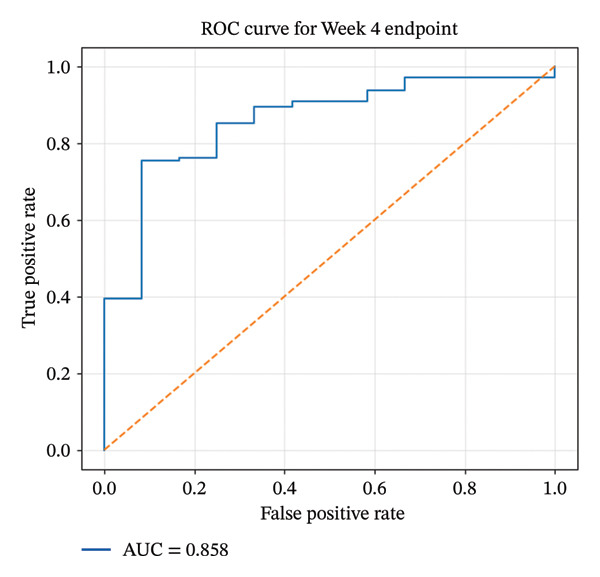
ROC curve at the 4‐week endpoint.

Figure 2 shows that there was substantial discrimination by the 4‐week model, with an out‐of‐fold ROC–AUC of 0.858.

**FIGURE 3:**
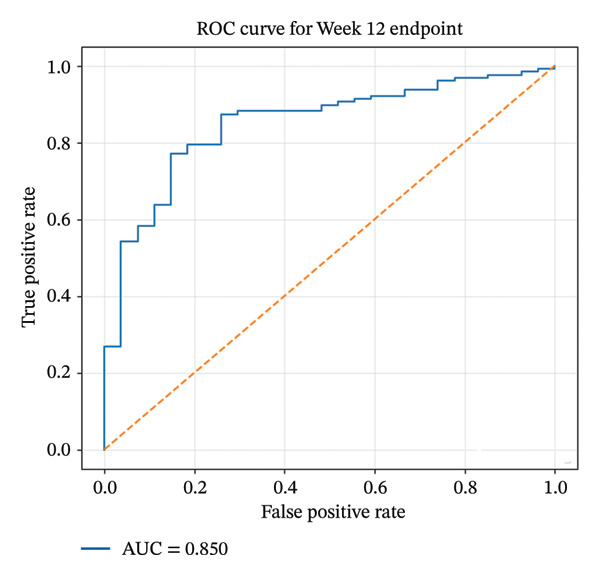
ROC curve at the 12‐week endpoint.

As per Figure 3, there was also excellent discrimination at 12 weeks, with an out‐of‐fold ROC–AUC of 0.850 and similar ROC curves.

**FIGURE 4:**
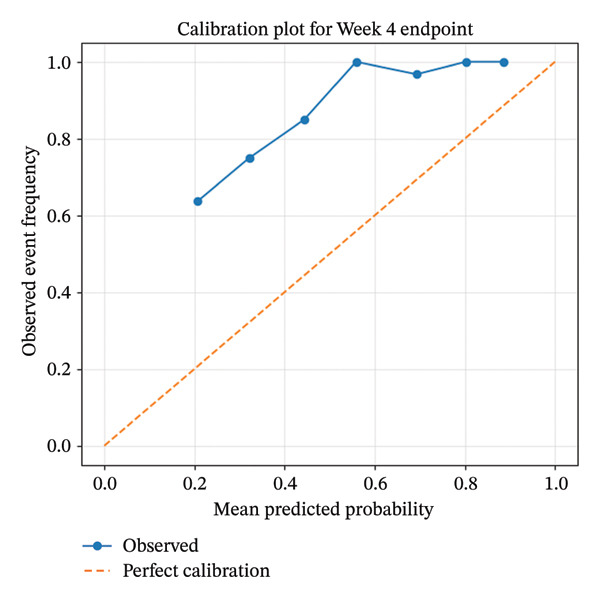
Calibration plot at the 4‐week endpoint.

Figure 4 illustrates that the calibration at the 4‐week mark was good, despite some deviation from the perfect line, which corresponds to a Brier score of 0.176.

**FIGURE 5:**
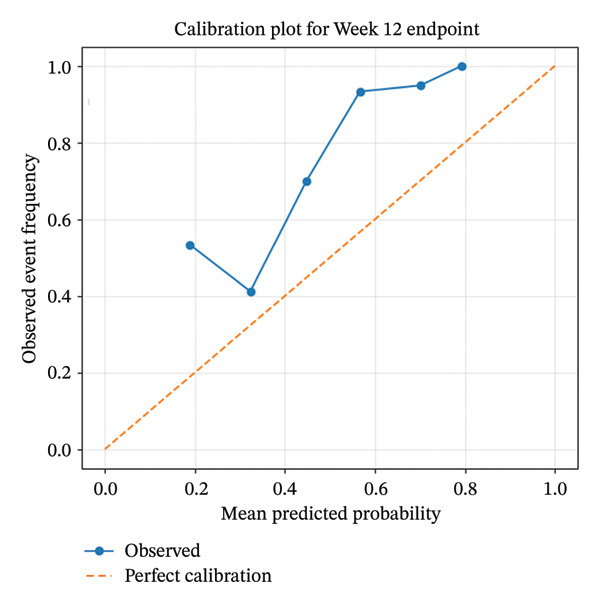
Calibration plot at the 12‐week endpoint.

As shown in Figure 5, there was relatively good calibration, and the probabilistic performance of the 12‐week model was a bit better than that of the 4‐week model as indicated by the lower Brier score of 0.174.

For the stratified hold‐out stability analysis performed on multiple occasions, at a training set fraction of 0.60 and 0.85, the results for the 4‐week endpoint demonstrated mean AUCs of 0.847 and 0.848, respectively, while their respective standard deviations were 0.061 and 0.128. On the other hand, for the 12‐week endpoint, mean AUCs of 0.836 and 0.830 were observed at training fractions of 0.60 and 0.85, and standard deviations of 0.058 and 0.101, respectively as shown in Table 5.

**TABLE 5:** Learning curve–style stability.

Endpoint	Training fraction	Mean training n	Mean training nonresponders	Mean AUC	SD AUC
Week 4	0.60	92	7	0.847	0.061
Week 4	0.85	130	10	0.848	0.128
Week 12	0.60	92	16	0.836	0.058
Week 12	0.85	130	23	0.830	0.101

The table states that the mean value for discrimination was fairly stable on repeated resamples but increased as the proportion trained became greater. The 4‐week endpoint continued to be more vulnerable owing to the limited minority cases.

**FIGURE 6:**
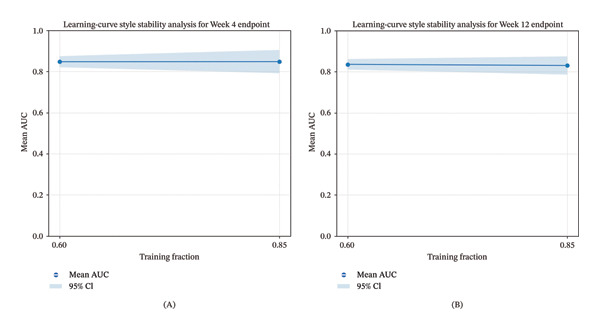
(A). Analysis of learning curve–style stability for the 4‐week endpoint. (B). Learning‐curve style stability test for the 12‐week endpoint.

The increased width of the confidence interval for a higher training fraction suggests an unstable model as shown in Figure 6A.

The 12‐week endpoint was more stable than the 4‐week endpoint. However, its uncertainty band expanded when the training fractions were high as shown in Figure 6B.

As shown in Table 6, HCV genotype 3 was a positive predictor at both follow‐up periods, while age, bilirubin, aminotransferases, and markers of fibrosis were negatively associated with response. These regression coefficients can only be seen as predictors but not causes of a response or lack thereof.

**TABLE 6:** Directional coefficients obtained from final logistic regression models.

Week 4 feature	Coefficient	Week 12 feature	Coefficient
HCV genotype 3	0.282	AST	−0.212
Age	−0.213	FIB‐4 score	−0.201
BMI, kg/m^2^	−0.191	ALT	−0.189
Treatment IFN naive	0.187	HCV genotype 3	0.181
HCV non‐genotype 3	−0.185	Age	−0.153
Hemoglobin	0.176	Hemoglobin	0.142
IL28 cc	0.164	Treatment IFN naïve	0.130
Bilirubin	−0.156	HCV‐RNA	−0.129
Male sex	0.137	Albumin	0.115
HCV‐RNA	−0.130	HBV‐DNA	−0.114

## 4. Discussion

Approximately 1.4 million deaths are related to hepatitis infection each year globally. Coinfections with HBV and HCV increase the risk of complications and difficulty in managing these cases. Figure 1 illustrates some common pathways between both viruses that help to determine variables for this study. Moreover, a study in Pakistan found that HCV causes chronic infection in about 70%–80% of people infected with both HCV and HBV [11].

The application of AI, more precisely ML techniques, has greatly influenced the field of healthcare, especially hepatology [12]. ML models have been shown to be successful at predicting responses to therapies using individual patient data such as viral genotype, fibrosis stage, and liver function tests [13]. In addition, ML algorithms can identify active antiviral drugs [14].

The present study used the leakage‐proof and internally validated LR pipeline for evaluating the effectiveness of therapy in patients with HBV–HCV coinfection after 4 and 12 weeks. A total of 154 patients who received the DAA therapy for 12 weeks and fulfilled the eligibility criteria were selected for the analysis. In the present study, various factors associated with the treatment were identified, such as Genotype 3 HCV, liver enzymes ALT and AST, and fibrosis markers FIB‐4 and APRI scores.

HCV genotype is a vital determinant of treatment effectiveness. Genotype 3 was found to have a significant relationship with better treatment response (*p* < 0.001). Out of all patients who exhibited good response to treatment, 85.9% were Genotype 3 patients, emphasizing the role played by this genotype in successful treatment. Similar findings have been made in previous studies where Genotype 3 patients have been shown to have positive responses toward treatment despite having high levels of HCV‐RNA [15]. There have also been reports where patients with Genotype 3 achieved SVRs of up to 95% using DAAs for 12 weeks [16].

FIB‐4 has been considered a reliable, noninvasive measure of assessing liver fibrosis, especially among those who are simultaneously infected with HBV/HCV [17]. Our findings show that there is a statistically significant relationship between high FIB‐4 scores and therapeutic response, at 4 and 12 weeks, respectively (*p* < 0.001). Responders had a mean FIB‐4 score of 1.77 ± 0.67, whereas the nonresponders had significantly higher FIB‐4 scores (2.43 ± 0.74). This suggests that higher FIB‐4 scores imply advanced liver fibrosis, which reduces the success rate of SVRs. Similar findings were reported showing a correlation between increased FIB‐4 scores and reduced success rate of treatment as well as fibrosis load [18].

The APRI score was incorporated along with the FIB‐4 score to determine the degree of liver fibrosis among patients with HBV/HCV coinfection after 4 and 12 weeks of treatment. It was observed that the APRI test results had significant differences (*p* < 0.001) between responders and nonresponders after 12 weeks, suggesting its suitability in determining the response status and assessing the degree of liver diseases. For instance, nonresponders were recorded to have relatively high mean APRI values of 0.97 ± 0.22. This corroborates the findings of Kabamba‐Tshikongo et al. [19] regarding the association between a high APRI score and liver fibrosis.

The enzyme tests related to liver function, specifically ALT and AST, were statistically different (*p* < 0.05) at both endpoints, which highlights the importance of ALT and AST in the determination of liver function. The result is supported by the findings of another study by Yeh et al. [20], showing that patients diagnosed with coinfection had similar outcomes, indicating that ALT and AST are important determinants of hepatic damage in viral hepatitis infections. This statement is further supported by a number of studies demonstrating that there are significant correlations between the increased levels of ALT (*p* = 0.046) and AST (*p* = 0.020) and liver inflammation among coinfected patients [21–23].

On the other hand, factors such as BMI (*p* = 0.052) were not statistically significant in determining the outcome of treatment during the period of 12 weeks. Even though BMI was nearly significant, still it could not cross the line of the statistical significance level (*p* < 0.05). Sex (*p* = 0.023), although marginally significant, was also not strong enough to infer a predictive value. At the 4‐week interval, other variables such as ALP (*p* = 0.303) and albumin (*p* = 0.523) also did not demonstrate statistical significance. Previous studies support the limited predictive utility of ALP and albumin in treatment monitoring because their fluctuations do not always reflect active hepatic pathology [24].

The LR technique was used for predicting the outcome based on data obtained prior to treatment at both time points. Figures 2 and 3 illustrate that, regardless of whether predictions were based on Week 4 or Week 12 samples, the developed model was able to consistently discriminate between positive and negative cases with ROC–AUC values of 0.858 and 0.850, respectively. According to Table 3, the performance at Week 12 was slightly better compared to that observed at Week 4, with increased accuracy (0.786 vs. 0.760), recall (0.772 vs. 0.746), and F1‐score (0.856 vs. 0.851) as well as a reduced Brier score (0.174 vs. 0.176), indicating better classification probability. ROC analysis performed at both endpoints confirmed excellent discrimination beyond the chance level, whereas the calibration plot at both weeks (Figures 4 and 5) indicated acceptable calibration, with relatively better calibration observed at Week 12. Although precision rates were remarkably high for both endpoints, there were more false negatives than false positives, indicating a conservative decision rule for classifying the positive case due to class imbalance. Such observations were similar to those of ML‐based cohort studies on predicting treatment outcomes by Park et al. [25] and Haga et al. [26], respectively.

Similar findings were reported by Al Alawi et al. [27], who used LR in mortality prediction in HCV infection and identified HBV coinfection as an important predictor (AUC = 0.929). While this study was aimed at HCV mortality prediction rather than the prediction of treatment outcomes, the discriminatory capacity shown in their model is consistent with that shown in the current research (AUC = 0.858 and 0.850).

**FIGURE 7:**
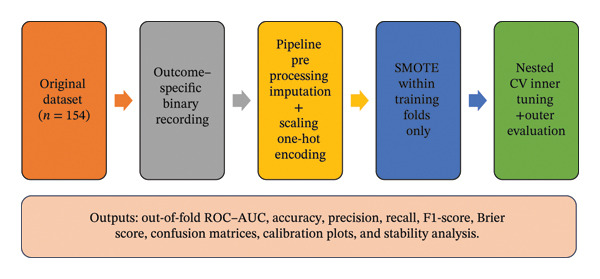
Leakage‐proof analytical workflow used for final confirmatory analyses.

One of the significant advantages of this study is the pipeline for the prevention of leakage (Figure 7), which provides separation of the processes of preprocessing of the data, use of SMOTE, and estimation of model performance to guarantee noncontamination of the data and increase trust in the results. Different approaches were used to evaluate model performance, such as the use of confusion matrices, ROC curves, and stability analysis with the help of bootstrapping, and calibration plots were used to increase the robustness of experiments.

There were some limitations in our study. First, the study took place only in one center. It is necessary to carry out further research involving other centers and more varied participants. Second, even with the application of nested cross‐validation, the study did not include any kind of external validation. Future studies should verify these models using other multicenter patient samples. Third, the relatively small sample size of 154 participants, along with the relatively few minority class occurrences, restricted the strength of the study and the sensitivity of the model at 4‐week intervals. Fourth, despite the use of the SMOTE technique for addressing the issue of class imbalance, the inherently small occurrence of events at 4 weeks is a fundamental flaw.

## 5. Future Perspective

Future studies could benefit from the use of techniques such as SHAP or LIME to facilitate explainability and usefulness in a clinical environment. Additionally, leveraging deep learning on well‐balanced larger datasets may uncover complex nonlinear relationships between predictors, which is something that LR cannot easily model without incorporating interactions or polynomial variables.

## 6. Conclusion

It is evident from this study that the use of a leakage‐free LR framework can be useful for predicting treatment outcomes among patients with coinfections of HBV and HCV. HCV genotype, FIB‐4, APRI, ALT, and AST were found to be significant predictors; HCV genotype 3 was associated with favorable treatment outcomes, whereas higher levels of fibrosis score and liver enzymes indicated poor responses to therapy. The proposed LR models exhibited good discriminative abilities, adequate calibration, and high consistency after 4 weeks and 12 weeks. Overall, the 12‐week LR model proved to be slightly better than the 4‐week model in both classification and probabilistic accuracy.

## Author Contributions

Concept, materials, data collection and processing, analysis, and interpretation: M.A. and J.A.; design: M.A., J.A., and M.A.M.; supervision: M.A.M. and M.A.; literature search and writing: A.M. and E.H.; critical review: M.A., M.A.M., A.M., and E.H.

## Funding

No funding was received for this manuscript.

## Conflicts of Interest

The authors declare no conflicts of interest.

## Supporting Information

Additional supporting information can be found online in the Supporting Information section.

## Supporting information


**Supporting Information 1** Supporting 1. Supplementary Material Supporting File S1: Jupyter notebook (Jupyter code 5) containing the full leakage‐proof analysis pipeline.

**Supporting Information 2** Supporting 2. Supporting Material Supporting File S2: Browser‐readable version of the python notebook file containing the full leakage‐proof analysis pipeline.

## Data Availability

The data that support the findings of this study are available on request from the corresponding author. The data are not publicly available due to privacy or ethical restrictions.
