# Supplementary material for: Prediction of Treatment Response in Hepatitis B and Hepatitis C Coinfected Patients Using a Leakage‐Proof, Internally Validated Logistic Regression Pipeline
Source: Adv Virol. 2026 Jul 9;2026:2578085. doi: 10.1155/av/2578085 (PMC13347315; doi:10.1155/av/2578085)
Supplement: Supplementary file 2 — Supporting Information 2 Supporting 2. Supporting Material Supporting File S2: Browser‐readable version of the python notebook file containing the full leakage‐proof analysis pipeline. [file AV-2026-2578085-s002.html]

Ayesha\_Maleha\_01042026


In [1]:

```
# Cell 1: environment and package check

import sys
import platform
import pandas as pd
import numpy as np
import matplotlib
import sklearn
import imblearn

print("Python version:", sys.version)
print("Platform:", platform.platform())
print("pandas:", pd.__version__)
print("numpy:", np.__version__)
print("matplotlib:", matplotlib.__version__)
print("scikit-learn:", sklearn.__version__)
print("imbalanced-learn:", imblearn.__version__)
```

```
Python version: 3.13.9 | packaged by Anaconda, Inc. | (main, Oct 21 2025, 19:09:58) [MSC v.1929 64 bit (AMD64)]
Platform: Windows-11-10.0.26200-SP0
pandas: 2.3.3
numpy: 2.3.5
matplotlib: 3.10.6
scikit-learn: 1.7.2
imbalanced-learn: 0.14.0
```

In [3]:

```
# Cell 2: load source dataset and verify structure

import pandas as pd

df = pd.read_csv("Maleha_HBV_HCV.csv")

print("Shape:", df.shape)
print("\nColumns:")
print(df.columns.tolist())

print("\nData types:")
print(df.dtypes)

print("\nFirst 5 rows:")
display(df.head())

print("\nMissing values per column:")
print(df.isna().sum())

print("\nDuplicate rows:", df.duplicated().sum())
```

```
Shape: (154, 18)

Columns:
['Post_treatment_4week', 'Post_treatment_12week', 'Albumin', 'Hemoglobin', 'gender', 'treatment_IFN', 'ALP', 'ALT', 'AST', 'HCV', 'HBV', 'APRI_score', 'FIB4_score', 'HCV_genotype', 'IL28_categorical', 'BMI', 'age_conti', 'Bilirubin']

Data types:
Post_treatment_4week      object
Post_treatment_12week     object
Albumin                  float64
Hemoglobin               float64
gender                    object
treatment_IFN             object
ALP                        int64
ALT                        int64
AST                        int64
HCV                      float64
HBV                      float64
APRI_score               float64
FIB4_score               float64
HCV_genotype              object
IL28_categorical          object
BMI                      float64
age_conti                  int64
Bilirubin                float64
dtype: object

First 5 rows:
```

|  | Post\_treatment\_4week | Post\_treatment\_12week | Albumin | Hemoglobin | gender | treatment\_IFN | ALP | ALT | AST | HCV | HBV | APRI\_score | FIB4\_score | HCV\_genotype | IL28\_categorical | BMI | age\_conti | Bilirubin |
| --- | --- | --- | --- | --- | --- | --- | --- | --- | --- | --- | --- | --- | --- | --- | --- | --- | --- | --- |
| 0 | responders | responder | 3.45 | 11.6 | male | naive | 91 | 18 | 20 | 2.45 | 4.50 | 0.80 | 1.62 | HCV genotype 3 | cc | 28.160430 | 34 | 1.02 |
| 1 | responders | responder | 4.56 | 11.0 | male | naive | 67 | 38 | 28 | 1.62 | 1.30 | 0.53 | 1.37 | HCV genotype 3 | cc | 24.891325 | 54 | 0.54 |
| 2 | responders | responder | 4.40 | 13.2 | female | naive | 76 | 64 | 57 | 2.40 | 1.00 | 0.70 | 2.12 | HCV genotype 3 | cc | 23.000000 | 53 | 1.22 |
| 3 | responders | responder | 4.50 | 15.3 | female | experienced | 102 | 80 | 45 | 5.62 | 4.76 | 0.90 | 2.91 | HCV genotype 3 | non-cc | 24.002568 | 46 | 1.19 |
| 4 | responders | responder | 4.42 | 14.1 | male | naive | 95 | 44 | 27 | 3.39 | 5.30 | 1.02 | 2.20 | HCV genotype 3 | cc | 22.563424 | 57 | 0.86 |

```
Missing values per column:
Post_treatment_4week     0
Post_treatment_12week    0
Albumin                  0
Hemoglobin               0
gender                   0
treatment_IFN            0
ALP                      0
ALT                      0
AST                      0
HCV                      0
HBV                      0
APRI_score               0
FIB4_score               0
HCV_genotype             0
IL28_categorical         0
BMI                      0
age_conti                0
Bilirubin                0
dtype: int64

Duplicate rows: 0
```

In [4]:

```
# Cell 3: verify outcome labels and class counts

print("4-week outcome labels and counts:")
print(df["Post_treatment_4week"].value_counts(dropna=False))

print("\n12-week outcome labels and counts:")
print(df["Post_treatment_12week"].value_counts(dropna=False))

print("\nUnique labels in 4-week outcome:")
print(df["Post_treatment_4week"].unique())

print("\nUnique labels in 12-week outcome:")
print(df["Post_treatment_12week"].unique())
```

```
4-week outcome labels and counts:
Post_treatment_4week
responders        142
non responders     12
Name: count, dtype: int64

12-week outcome labels and counts:
Post_treatment_12week
responder        127
non responder     27
Name: count, dtype: int64

Unique labels in 4-week outcome:
['responders' 'non responders']

Unique labels in 12-week outcome:
['responder' 'non responder']
```

In [5]:

```
# Cell 4: recode both outcomes into binary variables

df = df.copy()

df["y_4week"] = df["Post_treatment_4week"].map({
    "responders": 1,
    "non responders": 0
})

df["y_12week"] = df["Post_treatment_12week"].map({
    "responder": 1,
    "non responder": 0
})

print("Missing after 4-week recode:", df["y_4week"].isna().sum())
print("Missing after 12-week recode:", df["y_12week"].isna().sum())

print("\n4-week binary counts:")
print(df["y_4week"].value_counts().sort_index())

print("\n12-week binary counts:")
print(df["y_12week"].value_counts().sort_index())

print("\nPreview of original and recoded outcomes:")
display(df[[
    "Post_treatment_4week", "y_4week",
    "Post_treatment_12week", "y_12week"
]].head())
```

```
Missing after 4-week recode: 0
Missing after 12-week recode: 0

4-week binary counts:
y_4week
0     12
1    142
Name: count, dtype: int64

12-week binary counts:
y_12week
0     27
1    127
Name: count, dtype: int64

Preview of original and recoded outcomes:
```

|  | Post\_treatment\_4week | y\_4week | Post\_treatment\_12week | y\_12week |
| --- | --- | --- | --- | --- |
| 0 | responders | 1 | responder | 1 |
| 1 | responders | 1 | responder | 1 |
| 2 | responders | 1 | responder | 1 |
| 3 | responders | 1 | responder | 1 |
| 4 | responders | 1 | responder | 1 |

In [6]:

```
# Cell 5: define predictor sets and verify them

outcome_cols = [
    "Post_treatment_4week", "Post_treatment_12week",
    "y_4week", "y_12week"
]

numeric_features = [
    "Albumin", "Hemoglobin", "ALP", "ALT", "AST",
    "HCV", "HBV", "APRI_score", "FIB4_score",
    "BMI", "age_conti", "Bilirubin"
]

categorical_features = [
    "gender", "treatment_IFN", "HCV_genotype", "IL28_categorical"
]

all_predictors = numeric_features + categorical_features

print("Number of numeric predictors:", len(numeric_features))
print("Number of categorical predictors:", len(categorical_features))
print("Total predictors:", len(all_predictors))

print("\nNumeric predictors:")
print(numeric_features)

print("\nCategorical predictors:")
print(categorical_features)

print("\nAny predictor missing from dataframe?")
missing_predictors = [col for col in all_predictors if col not in df.columns]
print(missing_predictors)

print("\nPredictor-only dataframe shape:")
X_all = df[all_predictors].copy()
print(X_all.shape)

print("\nData types of predictor-only dataframe:")
print(X_all.dtypes)
```

```
Number of numeric predictors: 12
Number of categorical predictors: 4
Total predictors: 16

Numeric predictors:
['Albumin', 'Hemoglobin', 'ALP', 'ALT', 'AST', 'HCV', 'HBV', 'APRI_score', 'FIB4_score', 'BMI', 'age_conti', 'Bilirubin']

Categorical predictors:
['gender', 'treatment_IFN', 'HCV_genotype', 'IL28_categorical']

Any predictor missing from dataframe?
[]

Predictor-only dataframe shape:
(154, 16)

Data types of predictor-only dataframe:
Albumin             float64
Hemoglobin          float64
ALP                   int64
ALT                   int64
AST                   int64
HCV                 float64
HBV                 float64
APRI_score          float64
FIB4_score          float64
BMI                 float64
age_conti             int64
Bilirubin           float64
gender               object
treatment_IFN        object
HCV_genotype         object
IL28_categorical     object
dtype: object
```

In [7]:

```
# Cell 6: inspect categorical levels and basic numeric summaries

print("Categorical variable levels:\n")
for col in categorical_features:
    print(f"{col}:")
    print(df[col].value_counts(dropna=False))
    print("-" * 50)

print("\nNumeric summary:\n")
display(df[numeric_features].describe().T)

print("\nAny impossible or suspicious values check:")
checks = {
    "Albumin <= 0": (df["Albumin"] <= 0).sum(),
    "Hemoglobin <= 0": (df["Hemoglobin"] <= 0).sum(),
    "ALP <= 0": (df["ALP"] <= 0).sum(),
    "ALT <= 0": (df["ALT"] <= 0).sum(),
    "AST <= 0": (df["AST"] <= 0).sum(),
    "HCV < 0": (df["HCV"] < 0).sum(),
    "HBV < 0": (df["HBV"] < 0).sum(),
    "APRI_score < 0": (df["APRI_score"] < 0).sum(),
    "FIB4_score < 0": (df["FIB4_score"] < 0).sum(),
    "BMI <= 0": (df["BMI"] <= 0).sum(),
    "age_conti < 18": (df["age_conti"] < 18).sum(),
    "Bilirubin < 0": (df["Bilirubin"] < 0).sum(),
}

for k, v in checks.items():
    print(f"{k}: {v}")
```

```
Categorical variable levels:

gender:
gender
male      79
female    75
Name: count, dtype: int64
--------------------------------------------------
treatment_IFN:
treatment_IFN
naive          138
experienced     16
Name: count, dtype: int64
--------------------------------------------------
HCV_genotype:
HCV_genotype
HCV genotype 3        125
HCV non-genotype 3     29
Name: count, dtype: int64
--------------------------------------------------
IL28_categorical:
IL28_categorical
cc        106
non-cc     48
Name: count, dtype: int64
--------------------------------------------------

Numeric summary:
```

|  | count | mean | std | min | 25% | 50% | 75% | max |
| --- | --- | --- | --- | --- | --- | --- | --- | --- |
| Albumin | 154.0 | 4.301169 | 0.538507 | 3.20 | 3.9000 | 4.400000 | 4.700000 | 5.30 |
| Hemoglobin | 154.0 | 13.055714 | 1.537697 | 10.00 | 12.0000 | 12.900000 | 13.900000 | 17.60 |
| ALP | 154.0 | 80.785714 | 20.581407 | 43.00 | 66.2500 | 78.000000 | 96.000000 | 156.00 |
| ALT | 154.0 | 54.980519 | 19.382787 | 18.00 | 40.0000 | 53.500000 | 68.750000 | 108.00 |
| AST | 154.0 | 47.051948 | 23.944050 | 18.00 | 27.0000 | 42.000000 | 59.750000 | 112.00 |
| HCV | 154.0 | 2.732857 | 1.486827 | 1.13 | 1.5300 | 2.235000 | 3.640000 | 6.73 |
| HBV | 154.0 | 2.913701 | 1.717253 | 1.00 | 1.5000 | 2.300000 | 4.162500 | 7.97 |
| APRI\_score | 154.0 | 0.680881 | 0.354982 | 0.10 | 0.3800 | 0.645000 | 0.997500 | 1.43 |
| FIB4\_score | 154.0 | 1.822143 | 0.702373 | 0.98 | 1.2900 | 1.470000 | 2.370000 | 4.20 |
| BMI | 154.0 | 25.544826 | 2.512581 | 19.40 | 24.0000 | 25.225519 | 27.189947 | 31.00 |
| age\_conti | 154.0 | 50.753247 | 11.613780 | 20.00 | 42.0000 | 53.000000 | 60.000000 | 72.00 |
| Bilirubin | 154.0 | 0.817065 | 0.267323 | 0.33 | 0.5675 | 0.850000 | 1.000000 | 1.30 |

```
Any impossible or suspicious values check:
Albumin <= 0: 0
Hemoglobin <= 0: 0
ALP <= 0: 0
ALT <= 0: 0
AST <= 0: 0
HCV < 0: 0
HBV < 0: 0
APRI_score < 0: 0
FIB4_score < 0: 0
BMI <= 0: 0
age_conti < 18: 0
Bilirubin < 0: 0
```

In [8]:

```
# Cell 7: create endpoint-specific X and y objects

X = df[all_predictors].copy()
y4 = df["y_4week"].copy()
y12 = df["y_12week"].copy()

print("X shape:", X.shape)
print("y4 shape:", y4.shape)
print("y12 shape:", y12.shape)

print("\nFirst 5 rows of X:")
display(X.head())

print("\n4-week class counts:")
print(y4.value_counts().sort_index())

print("\n12-week class counts:")
print(y12.value_counts().sort_index())
```

```
X shape: (154, 16)
y4 shape: (154,)
y12 shape: (154,)

First 5 rows of X:
```

|  | Albumin | Hemoglobin | ALP | ALT | AST | HCV | HBV | APRI\_score | FIB4\_score | BMI | age\_conti | Bilirubin | gender | treatment\_IFN | HCV\_genotype | IL28\_categorical |
| --- | --- | --- | --- | --- | --- | --- | --- | --- | --- | --- | --- | --- | --- | --- | --- | --- |
| 0 | 3.45 | 11.6 | 91 | 18 | 20 | 2.45 | 4.50 | 0.80 | 1.62 | 28.160430 | 34 | 1.02 | male | naive | HCV genotype 3 | cc |
| 1 | 4.56 | 11.0 | 67 | 38 | 28 | 1.62 | 1.30 | 0.53 | 1.37 | 24.891325 | 54 | 0.54 | male | naive | HCV genotype 3 | cc |
| 2 | 4.40 | 13.2 | 76 | 64 | 57 | 2.40 | 1.00 | 0.70 | 2.12 | 23.000000 | 53 | 1.22 | female | naive | HCV genotype 3 | cc |
| 3 | 4.50 | 15.3 | 102 | 80 | 45 | 5.62 | 4.76 | 0.90 | 2.91 | 24.002568 | 46 | 1.19 | female | experienced | HCV genotype 3 | non-cc |
| 4 | 4.42 | 14.1 | 95 | 44 | 27 | 3.39 | 5.30 | 1.02 | 2.20 | 22.563424 | 57 | 0.86 | male | naive | HCV genotype 3 | cc |

```
4-week class counts:
y_4week
0     12
1    142
Name: count, dtype: int64

12-week class counts:
y_12week
0     27
1    127
Name: count, dtype: int64
```

In [9]:

```
# Cell 8: define preprocessing and modeling pipeline

from sklearn.compose import ColumnTransformer
from sklearn.pipeline import Pipeline as SkPipeline
from sklearn.impute import SimpleImputer
from sklearn.preprocessing import StandardScaler, OneHotEncoder
from sklearn.linear_model import LogisticRegression
from imblearn.pipeline import Pipeline as ImbPipeline
from imblearn.over_sampling import SMOTE

numeric_transformer = SkPipeline(steps=[
    ("imputer", SimpleImputer(strategy="median")),
    ("scaler", StandardScaler())
])

categorical_transformer = SkPipeline(steps=[
    ("imputer", SimpleImputer(strategy="most_frequent")),
    ("onehot", OneHotEncoder(handle_unknown="ignore"))
])

preprocessor = ColumnTransformer(
    transformers=[
        ("num", numeric_transformer, numeric_features),
        ("cat", categorical_transformer, categorical_features)
    ]
)

pipeline = ImbPipeline(steps=[
    ("preprocessor", preprocessor),
    ("smote", SMOTE(random_state=42)),
    ("model", LogisticRegression(
        max_iter=5000,
        solver="liblinear",
        random_state=42
    ))
])

print(pipeline)
```

```
Pipeline(steps=[('preprocessor',
                 ColumnTransformer(transformers=[('num',
                                                  Pipeline(steps=[('imputer',
                                                                   SimpleImputer(strategy='median')),
                                                                  ('scaler',
                                                                   StandardScaler())]),
                                                  ['Albumin', 'Hemoglobin',
                                                   'ALP', 'ALT', 'AST', 'HCV',
                                                   'HBV', 'APRI_score',
                                                   'FIB4_score', 'BMI',
                                                   'age_conti', 'Bilirubin']),
                                                 ('cat',
                                                  Pipeline(steps=[('imputer',
                                                                   SimpleImputer(strategy='most_frequent')),
                                                                  ('onehot',
                                                                   OneHotEncoder(handle_unknown='ignore'))]),
                                                  ['gender', 'treatment_IFN',
                                                   'HCV_genotype',
                                                   'IL28_categorical'])])),
                ('smote', SMOTE(random_state=42)),
                ('model',
                 LogisticRegression(max_iter=5000, random_state=42,
                                    solver='liblinear'))])
```

In [10]:

```
# Cell 9: define tuning grid and nested CV schemes

from sklearn.model_selection import StratifiedKFold

param_grid = {
    "smote__k_neighbors": [2, 3, 5],
    "model__C": [0.01, 0.1, 1, 10]
}

outer_cv_4 = StratifiedKFold(n_splits=5, shuffle=True, random_state=42)
inner_cv_4 = StratifiedKFold(n_splits=3, shuffle=True, random_state=42)

outer_cv_12 = StratifiedKFold(n_splits=3, shuffle=True, random_state=42)
inner_cv_12 = StratifiedKFold(n_splits=2, shuffle=True, random_state=42)

print("Parameter grid:")
print(param_grid)

print("\nWeek 4 CV:")
print("Outer folds =", outer_cv_4.get_n_splits())
print("Inner folds =", inner_cv_4.get_n_splits())

print("\nWeek 12 CV:")
print("Outer folds =", outer_cv_12.get_n_splits())
print("Inner folds =", inner_cv_12.get_n_splits())
```

```
Parameter grid:
{'smote__k_neighbors': [2, 3, 5], 'model__C': [0.01, 0.1, 1, 10]}

Week 4 CV:
Outer folds = 5
Inner folds = 3

Week 12 CV:
Outer folds = 3
Inner folds = 2
```

In [11]:

```
# Cell 10: import evaluation tools and define nested CV runner

import numpy as np
import pandas as pd

from sklearn.model_selection import GridSearchCV
from sklearn.metrics import (
    roc_auc_score,
    accuracy_score,
    precision_score,
    recall_score,
    f1_score,
    brier_score_loss,
    confusion_matrix,
    roc_curve
)

def run_nested_cv(X, y, pipeline, param_grid, outer_cv, inner_cv, endpoint_name="endpoint"):
    """
    Runs nested CV and returns:
    - out-of-fold predicted probabilities
    - out-of-fold predicted classes
    - fold-wise best parameters
    - summary metrics
    """
    y = pd.Series(y).reset_index(drop=True)
    X = X.reset_index(drop=True)

    oof_pred_proba = np.zeros(len(y), dtype=float)
    oof_pred_class = np.zeros(len(y), dtype=int)
    best_params_by_fold = []
    fold_summaries = []

    for fold, (train_idx, test_idx) in enumerate(outer_cv.split(X, y), start=1):
        X_train, X_test = X.iloc[train_idx], X.iloc[test_idx]
        y_train, y_test = y.iloc[train_idx], y.iloc[test_idx]

        grid = GridSearchCV(
            estimator=pipeline,
            param_grid=param_grid,
            scoring="roc_auc",
            cv=inner_cv,
            n_jobs=-1,
            refit=True
        )

        grid.fit(X_train, y_train)

        best_model = grid.best_estimator_
        probas = best_model.predict_proba(X_test)[:, 1]
        preds = (probas >= 0.5).astype(int)

        oof_pred_proba[test_idx] = probas
        oof_pred_class[test_idx] = preds

        fold_auc = roc_auc_score(y_test, probas)

        best_params_by_fold.append({
            "fold": fold,
            "best_params": grid.best_params_,
            "best_inner_cv_score": grid.best_score_
        })

        fold_summaries.append({
            "fold": fold,
            "n_train": len(train_idx),
            "n_test": len(test_idx),
            "train_positive": int(y_train.sum()),
            "train_negative": int((y_train == 0).sum()),
            "test_positive": int(y_test.sum()),
            "test_negative": int((y_test == 0).sum()),
            "fold_auc": fold_auc
        })

        print(f"{endpoint_name} | Fold {fold} done")
        print("  Best params:", grid.best_params_)
        print("  Inner CV AUC:", round(grid.best_score_, 4))
        print("  Outer fold AUC:", round(fold_auc, 4))

    tn, fp, fn, tp = confusion_matrix(y, oof_pred_class).ravel()

    metrics = {
        "N": len(y),
        "positive_class": int(y.sum()),
        "negative_class": int((y == 0).sum()),
        "oof_auc": roc_auc_score(y, oof_pred_proba),
        "accuracy": accuracy_score(y, oof_pred_class),
        "precision": precision_score(y, oof_pred_class, zero_division=0),
        "recall": recall_score(y, oof_pred_class, zero_division=0),
        "f1": f1_score(y, oof_pred_class, zero_division=0),
        "brier_score": brier_score_loss(y, oof_pred_proba),
        "TN": tn,
        "FP": fp,
        "FN": fn,
        "TP": tp
    }

    return {
        "oof_pred_proba": oof_pred_proba,
        "oof_pred_class": oof_pred_class,
        "best_params_by_fold": pd.DataFrame(best_params_by_fold),
        "fold_summaries": pd.DataFrame(fold_summaries),
        "metrics": metrics
    }

print("Nested CV function is ready.")
```

```
Nested CV function is ready.
```

In [12]:

```
# Cell 11: run nested CV for the 4-week endpoint

results_4 = run_nested_cv(
    X=X,
    y=y4,
    pipeline=pipeline,
    param_grid=param_grid,
    outer_cv=outer_cv_4,
    inner_cv=inner_cv_4,
    endpoint_name="Week 4"
)

print("\nWeek 4 overall metrics:")
for k, v in results_4["metrics"].items():
    if isinstance(v, float):
        print(f"{k}: {v:.4f}")
    else:
        print(f"{k}: {v}")

print("\nWeek 4 fold summaries:")
display(results_4["fold_summaries"])

print("\nWeek 4 best parameters by fold:")
display(results_4["best_params_by_fold"])
```

```
Week 4 | Fold 1 done
  Best params: {'model__C': 0.01, 'smote__k_neighbors': 3}
  Inner CV AUC: 0.8325
  Outer fold AUC: 0.9828
Week 4 | Fold 2 done
  Best params: {'model__C': 0.01, 'smote__k_neighbors': 3}
  Inner CV AUC: 0.8785
  Outer fold AUC: 0.931
Week 4 | Fold 3 done
  Best params: {'model__C': 0.01, 'smote__k_neighbors': 3}
  Inner CV AUC: 0.8363
  Outer fold AUC: 0.9167
Week 4 | Fold 4 done
  Best params: {'model__C': 0.01, 'smote__k_neighbors': 2}
  Inner CV AUC: 0.9123
  Outer fold AUC: 0.6905
Week 4 | Fold 5 done
  Best params: {'model__C': 0.01, 'smote__k_neighbors': 5}
  Inner CV AUC: 0.8202
  Outer fold AUC: 0.9286

Week 4 overall metrics:
N: 154
positive_class: 142
negative_class: 12
oof_auc: 0.8580
accuracy: 0.7597
precision: 0.9907
recall: 0.7465
f1: 0.8514
brier_score: 0.1761
TN: 11
FP: 1
FN: 36
TP: 106

Week 4 fold summaries:
```

|  | fold | n\_train | n\_test | train\_positive | train\_negative | test\_positive | test\_negative | fold\_auc |
| --- | --- | --- | --- | --- | --- | --- | --- | --- |
| 0 | 1 | 123 | 31 | 113 | 10 | 29 | 2 | 0.982759 |
| 1 | 2 | 123 | 31 | 113 | 10 | 29 | 2 | 0.931034 |
| 2 | 3 | 123 | 31 | 114 | 9 | 28 | 3 | 0.916667 |
| 3 | 4 | 123 | 31 | 114 | 9 | 28 | 3 | 0.690476 |
| 4 | 5 | 124 | 30 | 114 | 10 | 28 | 2 | 0.928571 |

```
Week 4 best parameters by fold:
```

|  | fold | best\_params | best\_inner\_cv\_score |
| --- | --- | --- | --- |
| 0 | 1 | {'model\_\_C': 0.01, 'smote\_\_k\_neighbors': 3} | 0.832543 |
| 1 | 2 | {'model\_\_C': 0.01, 'smote\_\_k\_neighbors': 3} | 0.878497 |
| 2 | 3 | {'model\_\_C': 0.01, 'smote\_\_k\_neighbors': 3} | 0.836257 |
| 3 | 4 | {'model\_\_C': 0.01, 'smote\_\_k\_neighbors': 2} | 0.912281 |
| 4 | 5 | {'model\_\_C': 0.01, 'smote\_\_k\_neighbors': 5} | 0.820175 |

In [13]:

```
# Cell 12: run nested CV for the 12-week endpoint

results_12 = run_nested_cv(
    X=X,
    y=y12,
    pipeline=pipeline,
    param_grid=param_grid,
    outer_cv=outer_cv_12,
    inner_cv=inner_cv_12,
    endpoint_name="Week 12"
)

print("\nWeek 12 overall metrics:")
for k, v in results_12["metrics"].items():
    if isinstance(v, float):
        print(f"{k}: {v:.4f}")
    else:
        print(f"{k}: {v}")

print("\nWeek 12 fold summaries:")
display(results_12["fold_summaries"])

print("\nWeek 12 best parameters by fold:")
display(results_12["best_params_by_fold"])
```

```
Week 12 | Fold 1 done
  Best params: {'model__C': 0.01, 'smote__k_neighbors': 3}
  Inner CV AUC: 0.7738
  Outer fold AUC: 0.8992
Week 12 | Fold 2 done
  Best params: {'model__C': 0.01, 'smote__k_neighbors': 2}
  Inner CV AUC: 0.8952
  Outer fold AUC: 0.8016
Week 12 | Fold 3 done
  Best params: {'model__C': 0.01, 'smote__k_neighbors': 2}
  Inner CV AUC: 0.8497
  Outer fold AUC: 0.8492

Week 12 overall metrics:
N: 154
positive_class: 127
negative_class: 27
oof_auc: 0.8495
accuracy: 0.7857
precision: 0.9608
recall: 0.7717
f1: 0.8559
brier_score: 0.1742
TN: 23
FP: 4
FN: 29
TP: 98

Week 12 fold summaries:
```

|  | fold | n\_train | n\_test | train\_positive | train\_negative | test\_positive | test\_negative | fold\_auc |
| --- | --- | --- | --- | --- | --- | --- | --- | --- |
| 0 | 1 | 102 | 52 | 84 | 18 | 43 | 9 | 0.899225 |
| 1 | 2 | 103 | 51 | 85 | 18 | 42 | 9 | 0.801587 |
| 2 | 3 | 103 | 51 | 85 | 18 | 42 | 9 | 0.849206 |

```
Week 12 best parameters by fold:
```

|  | fold | best\_params | best\_inner\_cv\_score |
| --- | --- | --- | --- |
| 0 | 1 | {'model\_\_C': 0.01, 'smote\_\_k\_neighbors': 3} | 0.773810 |
| 1 | 2 | {'model\_\_C': 0.01, 'smote\_\_k\_neighbors': 2} | 0.895226 |
| 2 | 3 | {'model\_\_C': 0.01, 'smote\_\_k\_neighbors': 2} | 0.849729 |

In [14]:

```
# Cell 13: create a combined performance summary table

summary_table = pd.DataFrame([
    {
        "Endpoint": "Week 4",
        "N": results_4["metrics"]["N"],
        "Positive class": results_4["metrics"]["positive_class"],
        "Negative class": results_4["metrics"]["negative_class"],
        "OOF AUC": results_4["metrics"]["oof_auc"],
        "Accuracy": results_4["metrics"]["accuracy"],
        "Precision": results_4["metrics"]["precision"],
        "Recall": results_4["metrics"]["recall"],
        "F1": results_4["metrics"]["f1"],
        "Brier score": results_4["metrics"]["brier_score"],
        "TN": results_4["metrics"]["TN"],
        "FP": results_4["metrics"]["FP"],
        "FN": results_4["metrics"]["FN"],
        "TP": results_4["metrics"]["TP"],
    },
    {
        "Endpoint": "Week 12",
        "N": results_12["metrics"]["N"],
        "Positive class": results_12["metrics"]["positive_class"],
        "Negative class": results_12["metrics"]["negative_class"],
        "OOF AUC": results_12["metrics"]["oof_auc"],
        "Accuracy": results_12["metrics"]["accuracy"],
        "Precision": results_12["metrics"]["precision"],
        "Recall": results_12["metrics"]["recall"],
        "F1": results_12["metrics"]["f1"],
        "Brier score": results_12["metrics"]["brier_score"],
        "TN": results_12["metrics"]["TN"],
        "FP": results_12["metrics"]["FP"],
        "FN": results_12["metrics"]["FN"],
        "TP": results_12["metrics"]["TP"],
    }
])

summary_table_rounded = summary_table.copy()
for col in ["OOF AUC", "Accuracy", "Precision", "Recall", "F1", "Brier score"]:
    summary_table_rounded[col] = summary_table_rounded[col].round(4)

display(summary_table_rounded)
```

|  | Endpoint | N | Positive class | Negative class | OOF AUC | Accuracy | Precision | Recall | F1 | Brier score | TN | FP | FN | TP |
| --- | --- | --- | --- | --- | --- | --- | --- | --- | --- | --- | --- | --- | --- | --- |
| 0 | Week 4 | 154 | 142 | 12 | 0.8580 | 0.7597 | 0.9907 | 0.7465 | 0.8514 | 0.1761 | 11 | 1 | 36 | 106 |
| 1 | Week 12 | 154 | 127 | 27 | 0.8495 | 0.7857 | 0.9608 | 0.7717 | 0.8559 | 0.1742 | 23 | 4 | 29 | 98 |

In [15]:

```
# Cell 14: bootstrap 95% CI for OOF AUC

from sklearn.metrics import roc_auc_score

def bootstrap_auc_ci(y_true, y_proba, n_bootstrap=2000, random_state=42):
    rng = np.random.default_rng(random_state)
    y_true = np.asarray(y_true)
    y_proba = np.asarray(y_proba)

    aucs = []
    n = len(y_true)

    for _ in range(n_bootstrap):
        idx = rng.integers(0, n, n)
        y_b = y_true[idx]
        p_b = y_proba[idx]

        # Need both classes present for ROC-AUC
        if len(np.unique(y_b)) < 2:
            continue

        aucs.append(roc_auc_score(y_b, p_b))

    aucs = np.array(aucs)
    lower = np.percentile(aucs, 2.5)
    upper = np.percentile(aucs, 97.5)
    mean_auc = np.mean(aucs)

    return {
        "bootstrap_mean_auc": mean_auc,
        "ci_lower": lower,
        "ci_upper": upper,
        "n_valid_bootstraps": len(aucs)
    }

ci_4 = bootstrap_auc_ci(y4.values, results_4["oof_pred_proba"], n_bootstrap=2000, random_state=42)
ci_12 = bootstrap_auc_ci(y12.values, results_12["oof_pred_proba"], n_bootstrap=2000, random_state=42)

print("Week 4 AUC CI:")
for k, v in ci_4.items():
    if isinstance(v, float):
        print(f"{k}: {v:.4f}")
    else:
        print(f"{k}: {v}")

print("\nWeek 12 AUC CI:")
for k, v in ci_12.items():
    if isinstance(v, float):
        print(f"{k}: {v:.4f}")
    else:
        print(f"{k}: {v}")
```

```
Week 4 AUC CI:
bootstrap_mean_auc: 0.8558
ci_lower: 0.7456
ci_upper: 0.9418
n_valid_bootstraps: 2000

Week 12 AUC CI:
bootstrap_mean_auc: 0.8482
ci_lower: 0.7690
ci_upper: 0.9159
n_valid_bootstraps: 2000
```

In [16]:

```
# Cell 15: ROC curves for week 4 and week 12

import matplotlib.pyplot as plt
from sklearn.metrics import roc_curve, roc_auc_score

# Week 4 ROC
fpr_4, tpr_4, _ = roc_curve(y4, results_4["oof_pred_proba"])
auc_4 = roc_auc_score(y4, results_4["oof_pred_proba"])

plt.figure(figsize=(7, 6))
plt.plot(fpr_4, tpr_4, label=f"AUC = {auc_4:.3f}")
plt.plot([0, 1], [0, 1], linestyle="--")
plt.xlabel("False Positive Rate")
plt.ylabel("True Positive Rate")
plt.title("ROC Curve for Week 4 Endpoint")
plt.legend(loc="lower right")
plt.grid(True, alpha=0.3)
plt.show()

# Week 12 ROC
fpr_12, tpr_12, _ = roc_curve(y12, results_12["oof_pred_proba"])
auc_12 = roc_auc_score(y12, results_12["oof_pred_proba"])

plt.figure(figsize=(7, 6))
plt.plot(fpr_12, tpr_12, label=f"AUC = {auc_12:.3f}")
plt.plot([0, 1], [0, 1], linestyle="--")
plt.xlabel("False Positive Rate")
plt.ylabel("True Positive Rate")
plt.title("ROC Curve for Week 12 Endpoint")
plt.legend(loc="lower right")
plt.grid(True, alpha=0.3)
plt.show()
```

In [17]:

```
# Cell 16: calibration plots for week 4 and week 12

import matplotlib.pyplot as plt
from sklearn.calibration import calibration_curve

# Week 4 calibration
prob_true_4, prob_pred_4 = calibration_curve(
    y4, results_4["oof_pred_proba"], n_bins=8, strategy="uniform"
)

plt.figure(figsize=(7, 6))
plt.plot(prob_pred_4, prob_true_4, marker="o", label="Observed")
plt.plot([0, 1], [0, 1], linestyle="--", label="Perfect calibration")
plt.xlabel("Mean predicted probability")
plt.ylabel("Observed event frequency")
plt.title("Calibration Plot for Week 4 Endpoint")
plt.legend(loc="upper left")
plt.grid(True, alpha=0.3)
plt.show()

# Week 12 calibration
prob_true_12, prob_pred_12 = calibration_curve(
    y12, results_12["oof_pred_proba"], n_bins=8, strategy="uniform"
)

plt.figure(figsize=(7, 6))
plt.plot(prob_pred_12, prob_true_12, marker="o", label="Observed")
plt.plot([0, 1], [0, 1], linestyle="--", label="Perfect calibration")
plt.xlabel("Mean predicted probability")
plt.ylabel("Observed event frequency")
plt.title("Calibration Plot for Week 12 Endpoint")
plt.legend(loc="upper left")
plt.grid(True, alpha=0.3)
plt.show()
```

In [20]:

```
# Cell 17 (safer): learning-curve style stability analysis for sparse-event data

from sklearn.model_selection import StratifiedShuffleSplit, StratifiedKFold, GridSearchCV
from sklearn.metrics import roc_auc_score
from sklearn.base import clone
import numpy as np
import pandas as pd

# Ultra-safe grid for sparse minority settings
stability_param_grid = {
    "smote__k_neighbors": [1, 2],
    "model__C": [0.01, 0.1, 1, 10]
}

def learning_curve_stability(X, y, pipeline, param_grid, train_sizes=(0.60, 0.85), n_repeats=20, random_state=42):
    rows = []

    for train_frac in train_sizes:
        splitter = StratifiedShuffleSplit(
            n_splits=n_repeats,
            train_size=train_frac,
            random_state=random_state
        )

        aucs = []
        train_ns = []
        train_negs = []

        for split_id, (train_idx, test_idx) in enumerate(splitter.split(X, y), start=1):
            X_train, X_test = X.iloc[train_idx], X.iloc[test_idx]
            y_train, y_test = y.iloc[train_idx], y.iloc[test_idx]

            # With very sparse classes, keep inner CV minimal
            inner_cv = StratifiedKFold(n_splits=2, shuffle=True, random_state=42)

            grid = GridSearchCV(
                estimator=clone(pipeline),
                param_grid=param_grid,
                scoring="roc_auc",
                cv=inner_cv,
                n_jobs=1,
                refit=True,
                error_score="raise"
            )

            grid.fit(X_train, y_train)
            y_proba = grid.best_estimator_.predict_proba(X_test)[:, 1]
            auc = roc_auc_score(y_test, y_proba)

            aucs.append(auc)
            train_ns.append(len(train_idx))
            train_negs.append(int((y_train == 0).sum()))

        rows.append({
            "Training fraction": train_frac,
            "Mean training n": int(round(np.mean(train_ns))),
            "Mean training non-responders": int(round(np.mean(train_negs))),
            "Mean AUC": float(np.mean(aucs)),
            "SD AUC": float(np.std(aucs, ddof=1)),
            "All AUCs": aucs
        })

    return pd.DataFrame(rows)

stability_4 = learning_curve_stability(
    X=X,
    y=y4,
    pipeline=pipeline,
    param_grid=stability_param_grid,
    train_sizes=(0.60, 0.85),
    n_repeats=20,
    random_state=42
)

stability_12 = learning_curve_stability(
    X=X,
    y=y12,
    pipeline=pipeline,
    param_grid=stability_param_grid,
    train_sizes=(0.60, 0.85),
    n_repeats=20,
    random_state=42
)

print("Week 4 stability summary:")
display(stability_4[["Training fraction", "Mean training n", "Mean training non-responders", "Mean AUC", "SD AUC"]])

print("\nWeek 12 stability summary:")
display(stability_12[["Training fraction", "Mean training n", "Mean training non-responders", "Mean AUC", "SD AUC"]])
```

```
Week 4 stability summary:
```

|  | Training fraction | Mean training n | Mean training non-responders | Mean AUC | SD AUC |
| --- | --- | --- | --- | --- | --- |
| 0 | 0.60 | 92 | 7 | 0.847368 | 0.060864 |
| 1 | 0.85 | 130 | 10 | 0.847727 | 0.128374 |

```
Week 12 stability summary:
```

|  | Training fraction | Mean training n | Mean training non-responders | Mean AUC | SD AUC |
| --- | --- | --- | --- | --- | --- |
| 0 | 0.60 | 92 | 16 | 0.835651 | 0.058140 |
| 1 | 0.85 | 130 | 23 | 0.830000 | 0.100933 |

In [22]:

```
# Cell 18 (improved): stability plots with 95% CI shading

import numpy as np
import matplotlib.pyplot as plt

n_repeats = 20  # same as used in the stability analysis

def add_ci_columns(stability_df, n_repeats=20):
    df_plot = stability_df.copy()
    df_plot["SE"] = df_plot["SD AUC"] / np.sqrt(n_repeats)
    df_plot["CI lower"] = df_plot["Mean AUC"] - 1.96 * df_plot["SE"]
    df_plot["CI upper"] = df_plot["Mean AUC"] + 1.96 * df_plot["SE"]
    return df_plot

stability_4_plot = add_ci_columns(stability_4, n_repeats=n_repeats)
stability_12_plot = add_ci_columns(stability_12, n_repeats=n_repeats)

# Week 4
x4 = stability_4_plot["Training fraction"].to_numpy(dtype=float)
y4_mean = stability_4_plot["Mean AUC"].to_numpy(dtype=float)
y4_low = stability_4_plot["CI lower"].to_numpy(dtype=float)
y4_high = stability_4_plot["CI upper"].to_numpy(dtype=float)

plt.figure(figsize=(7, 6))
plt.plot(x4, y4_mean, marker="o", label="Mean AUC")
plt.fill_between(x4, y4_low, y4_high, alpha=0.20, label="95% CI")
plt.xlabel("Training fraction")
plt.ylabel("Mean AUC")
plt.title("Learning-Curve Style Stability Analysis for Week 4 Endpoint")
plt.xticks(x4)
plt.ylim(0.0, 1.0)
plt.grid(True, alpha=0.3)
plt.legend()
plt.show()

# Week 12
x12 = stability_12_plot["Training fraction"].to_numpy(dtype=float)
y12_mean = stability_12_plot["Mean AUC"].to_numpy(dtype=float)
y12_low = stability_12_plot["CI lower"].to_numpy(dtype=float)
y12_high = stability_12_plot["CI upper"].to_numpy(dtype=float)

plt.figure(figsize=(7, 6))
plt.plot(x12, y12_mean, marker="o", label="Mean AUC")
plt.fill_between(x12, y12_low, y12_high, alpha=0.20, label="95% CI")
plt.xlabel("Training fraction")
plt.ylabel("Mean AUC")
plt.title("Learning-Curve Style Stability Analysis for Week 12 Endpoint")
plt.xticks(x12)
plt.ylim(0.0, 1.0)
plt.grid(True, alpha=0.3)
plt.legend()
plt.show()

print("Week 4 plotting table:")
display(stability_4_plot[["Training fraction", "Mean AUC", "SD AUC", "SE", "CI lower", "CI upper"]])

print("\nWeek 12 plotting table:")
display(stability_12_plot[["Training fraction", "Mean AUC", "SD AUC", "SE", "CI lower", "CI upper"]])
```

```
Week 4 plotting table:
```

|  | Training fraction | Mean AUC | SD AUC | SE | CI lower | CI upper |
| --- | --- | --- | --- | --- | --- | --- |
| 0 | 0.60 | 0.847368 | 0.060864 | 0.013610 | 0.820693 | 0.874043 |
| 1 | 0.85 | 0.847727 | 0.128374 | 0.028705 | 0.791465 | 0.903990 |

```
Week 12 plotting table:
```

|  | Training fraction | Mean AUC | SD AUC | SE | CI lower | CI upper |
| --- | --- | --- | --- | --- | --- | --- |
| 0 | 0.60 | 0.835651 | 0.058140 | 0.013001 | 0.810169 | 0.861132 |
| 1 | 0.85 | 0.830000 | 0.100933 | 0.022569 | 0.785764 | 0.874236 |

In [23]:

```
# Cell 19: fit final full-data models and extract coefficients

from sklearn.base import clone

def fit_final_model_and_get_coefficients(X, y, pipeline, best_C=0.01, best_k=2):
    final_model = clone(pipeline)
    final_model.set_params(
        model__C=best_C,
        smote__k_neighbors=best_k
    )
    final_model.fit(X, y)

    # Get transformed feature names
    preprocessor_fitted = final_model.named_steps["preprocessor"]
    feature_names = preprocessor_fitted.get_feature_names_out()

    # Logistic regression coefficients
    coef = final_model.named_steps["model"].coef_[0]

    coef_df = pd.DataFrame({
        "feature": feature_names,
        "coefficient": coef,
        "abs_coefficient": np.abs(coef)
    }).sort_values("abs_coefficient", ascending=False).reset_index(drop=True)

    return final_model, coef_df

# Use the most commonly selected settings from the CV runs
final_model_4, coef_4 = fit_final_model_and_get_coefficients(
    X=X, y=y4, pipeline=pipeline, best_C=0.01, best_k=3
)

final_model_12, coef_12 = fit_final_model_and_get_coefficients(
    X=X, y=y12, pipeline=pipeline, best_C=0.01, best_k=2
)

print("Top coefficients for Week 4 model:")
display(coef_4.head(15))

print("\nTop coefficients for Week 12 model:")
display(coef_12.head(15))
```

```
Top coefficients for Week 4 model:
```

|  | feature | coefficient | abs\_coefficient |
| --- | --- | --- | --- |
| 0 | cat\_\_HCV\_genotype\_HCV genotype 3 | 0.282111 | 0.282111 |
| 1 | num\_\_age\_conti | -0.213382 | 0.213382 |
| 2 | num\_\_BMI | -0.191133 | 0.191133 |
| 3 | cat\_\_treatment\_IFN\_naive | 0.187185 | 0.187185 |
| 4 | cat\_\_HCV\_genotype\_HCV non-genotype 3 | -0.185028 | 0.185028 |
| 5 | num\_\_Hemoglobin | 0.176160 | 0.176160 |
| 6 | cat\_\_IL28\_categorical\_cc | 0.164068 | 0.164068 |
| 7 | num\_\_Bilirubin | -0.155509 | 0.155509 |
| 8 | cat\_\_gender\_male | 0.137194 | 0.137194 |
| 9 | num\_\_HCV | -0.130019 | 0.130019 |
| 10 | num\_\_APRI\_score | -0.127569 | 0.127569 |
| 11 | num\_\_AST | -0.117507 | 0.117507 |
| 12 | num\_\_HBV | -0.097236 | 0.097236 |
| 13 | num\_\_ALT | -0.096304 | 0.096304 |
| 14 | num\_\_FIB4\_score | -0.094298 | 0.094298 |

```
Top coefficients for Week 12 model:
```

|  | feature | coefficient | abs\_coefficient |
| --- | --- | --- | --- |
| 0 | num\_\_AST | -0.212214 | 0.212214 |
| 1 | num\_\_FIB4\_score | -0.201388 | 0.201388 |
| 2 | num\_\_ALT | -0.188801 | 0.188801 |
| 3 | cat\_\_HCV\_genotype\_HCV genotype 3 | 0.180789 | 0.180789 |
| 4 | num\_\_age\_conti | -0.153047 | 0.153047 |
| 5 | num\_\_Hemoglobin | 0.142472 | 0.142472 |
| 6 | cat\_\_treatment\_IFN\_naive | 0.129819 | 0.129819 |
| 7 | num\_\_HCV | -0.129016 | 0.129016 |
| 8 | num\_\_Albumin | 0.115028 | 0.115028 |
| 9 | num\_\_HBV | -0.114062 | 0.114062 |
| 10 | num\_\_APRI\_score | -0.110249 | 0.110249 |
| 11 | cat\_\_HCV\_genotype\_HCV non-genotype 3 | -0.107471 | 0.107471 |
| 12 | cat\_\_IL28\_categorical\_cc | 0.106687 | 0.106687 |
| 13 | cat\_\_gender\_male | 0.085296 | 0.085296 |
| 14 | num\_\_Bilirubin | -0.062167 | 0.062167 |

In [24]:

```
# Cell 20: export summary tables for manuscript drafting

# AUC CI table
auc_ci_table = pd.DataFrame([
    {
        "Endpoint": "Week 4",
        "OOF AUC": round(results_4["metrics"]["oof_auc"], 4),
        "CI lower": round(ci_4["ci_lower"], 4),
        "CI upper": round(ci_4["ci_upper"], 4),
        "Bootstrap mean AUC": round(ci_4["bootstrap_mean_auc"], 4),
        "Valid bootstraps": ci_4["n_valid_bootstraps"]
    },
    {
        "Endpoint": "Week 12",
        "OOF AUC": round(results_12["metrics"]["oof_auc"], 4),
        "CI lower": round(ci_12["ci_lower"], 4),
        "CI upper": round(ci_12["ci_upper"], 4),
        "Bootstrap mean AUC": round(ci_12["bootstrap_mean_auc"], 4),
        "Valid bootstraps": ci_12["n_valid_bootstraps"]
    }
])

# Stability tables with CI shading values
stability_4_export = stability_4_plot.copy()
stability_4_export["Endpoint"] = "Week 4"

stability_12_export = stability_12_plot.copy()
stability_12_export["Endpoint"] = "Week 12"

stability_export = pd.concat([stability_4_export, stability_12_export], ignore_index=True)

# Save to Excel
with pd.ExcelWriter("HBV_HCV_analysis_outputs.xlsx", engine="openpyxl") as writer:
    summary_table_rounded.to_excel(writer, sheet_name="Performance Summary", index=False)
    auc_ci_table.to_excel(writer, sheet_name="AUC 95CI", index=False)
    results_4["fold_summaries"].to_excel(writer, sheet_name="Week4 Fold Summaries", index=False)
    results_12["fold_summaries"].to_excel(writer, sheet_name="Week12 Fold Summaries", index=False)
    results_4["best_params_by_fold"].to_excel(writer, sheet_name="Week4 Best Params", index=False)
    results_12["best_params_by_fold"].to_excel(writer, sheet_name="Week12 Best Params", index=False)
    coef_4.to_excel(writer, sheet_name="Week4 Coefficients", index=False)
    coef_12.to_excel(writer, sheet_name="Week12 Coefficients", index=False)
    stability_export.to_excel(writer, sheet_name="Stability Summary", index=False)

# Save key CSV files too
summary_table_rounded.to_csv("performance_summary.csv", index=False)
auc_ci_table.to_csv("auc_ci_table.csv", index=False)
coef_4.to_csv("week4_coefficients.csv", index=False)
coef_12.to_csv("week12_coefficients.csv", index=False)
stability_export.to_csv("stability_summary.csv", index=False)

print("Files exported successfully:")
print("- HBV_HCV_analysis_outputs.xlsx")
print("- performance_summary.csv")
print("- auc_ci_table.csv")
print("- week4_coefficients.csv")
print("- week12_coefficients.csv")
print("- stability_summary.csv")
```

```
Files exported successfully:
- HBV_HCV_analysis_outputs.xlsx
- performance_summary.csv
- auc_ci_table.csv
- week4_coefficients.csv
- week12_coefficients.csv
- stability_summary.csv
```

In [25]:

```
# Cell 21: print compact manuscript-ready results text

week4_text = (
    f"At 4 weeks, the internally validated logistic regression pipeline achieved an "
    f"out-of-fold AUC of {results_4['metrics']['oof_auc']:.3f} "
    f"(95% CI {ci_4['ci_lower']:.3f} to {ci_4['ci_upper']:.3f}), "
    f"with accuracy {results_4['metrics']['accuracy']:.3f}, "
    f"precision {results_4['metrics']['precision']:.3f}, "
    f"recall {results_4['metrics']['recall']:.3f}, "
    f"F1-score {results_4['metrics']['f1']:.3f}, and "
    f"Brier score {results_4['metrics']['brier_score']:.3f}. "
    f"The aggregated confusion matrix showed TN={results_4['metrics']['TN']}, "
    f"FP={results_4['metrics']['FP']}, FN={results_4['metrics']['FN']}, "
    f"and TP={results_4['metrics']['TP']}."
)

week12_text = (
    f"At 12 weeks, the internally validated logistic regression pipeline achieved an "
    f"out-of-fold AUC of {results_12['metrics']['oof_auc']:.3f} "
    f"(95% CI {ci_12['ci_lower']:.3f} to {ci_12['ci_upper']:.3f}), "
    f"with accuracy {results_12['metrics']['accuracy']:.3f}, "
    f"precision {results_12['metrics']['precision']:.3f}, "
    f"recall {results_12['metrics']['recall']:.3f}, "
    f"F1-score {results_12['metrics']['f1']:.3f}, and "
    f"Brier score {results_12['metrics']['brier_score']:.3f}. "
    f"The aggregated confusion matrix showed TN={results_12['metrics']['TN']}, "
    f"FP={results_12['metrics']['FP']}, FN={results_12['metrics']['FN']}, "
    f"and TP={results_12['metrics']['TP']}."
)

stability_text = (
    f"In repeated stratified holdout stability analyses, the 4-week endpoint showed "
    f"mean AUCs of {stability_4_plot.loc[0, 'Mean AUC']:.3f} "
    f"and {stability_4_plot.loc[1, 'Mean AUC']:.3f} at training fractions of 0.60 "
    f"and 0.85, with corresponding standard deviations of "
    f"{stability_4_plot.loc[0, 'SD AUC']:.3f} and {stability_4_plot.loc[1, 'SD AUC']:.3f}. "
    f"For the 12-week endpoint, the corresponding mean AUCs were "
    f"{stability_12_plot.loc[0, 'Mean AUC']:.3f} and {stability_12_plot.loc[1, 'Mean AUC']:.3f}, "
    f"with standard deviations of {stability_12_plot.loc[0, 'SD AUC']:.3f} "
    f"and {stability_12_plot.loc[1, 'SD AUC']:.3f}."
)

print("Results paragraph 1:\n")
print(week4_text)

print("\nResults paragraph 2:\n")
print(week12_text)

print("\nResults paragraph 3:\n")
print(stability_text)
```

```
Results paragraph 1:

At 4 weeks, the internally validated logistic regression pipeline achieved an out-of-fold AUC of 0.858 (95% CI 0.746 to 0.942), with accuracy 0.760, precision 0.991, recall 0.746, F1-score 0.851, and Brier score 0.176. The aggregated confusion matrix showed TN=11, FP=1, FN=36, and TP=106.

Results paragraph 2:

At 12 weeks, the internally validated logistic regression pipeline achieved an out-of-fold AUC of 0.850 (95% CI 0.769 to 0.916), with accuracy 0.786, precision 0.961, recall 0.772, F1-score 0.856, and Brier score 0.174. The aggregated confusion matrix showed TN=23, FP=4, FN=29, and TP=98.

Results paragraph 3:

In repeated stratified holdout stability analyses, the 4-week endpoint showed mean AUCs of 0.847 and 0.848 at training fractions of 0.60 and 0.85, with corresponding standard deviations of 0.061 and 0.128. For the 12-week endpoint, the corresponding mean AUCs were 0.836 and 0.830, with standard deviations of 0.058 and 0.101.
```

In [ ]:

```

```
